# Effects of NSC in different organs and at different growth stages on the yield of oil peony Fengdan with different ages

**DOI:** 10.3389/fpls.2023.1108668

**Published:** 2023-04-14

**Authors:** Chengzhong Wang, Xiaoyi Ma, Qingkui Li, Yonghong Hu, Ji Yang, Zhiping Song

**Affiliations:** ^1^ The Ministry of Education Key Laboratory for Biodiversity Science and Ecological Engineering, Institute of Biodiversity Science, National Observations and Research Station for Wetland Ecosystems of the Yangtze Estuary, Fudan University, Shanghai, China; ^2^ Institute of Botany, National Observations and Research Station for Wetland Ecosystems of the Yangtze Estuary, Fudan University, Shanghai, China; ^3^ College of Horticultural Science and Technology, Suzhou Polytechnic Institute of Agriculture, Suzhou, China; ^4^ Shanghai Key Laboratory of Plant Functional Genomics and Resources, Shanghai Chenshan Botanical Garden, Shanghai, China

**Keywords:** age effect, biomass allocation, crop yield, non-structural carbohydrates, soluble sugars, starch, oil peony, *Paeonia ostii*

## Abstract

Non-structural carbohydrates (NSC) as resource reserves of plants play important roles in energy supply for normal growth and reproduction under environmental stress. The yield of perennial crops is mainly determined by the carbohydrate production and allocation in the current growth season, as well as the re-allocation of NSC reserves. However, the contribution of NSC to crop yield has not been fully determined. Fengdan (*Paeonia ostii*) is a variety of oil Peony that is newly developed in China. The effects of tree age and NSC on yield were examined by investigated the variations of biomass, soluble sugars, starch, and NSC in the organ and whole tree levels in the dormant and ripening stages of Fengdan populations with 4-, 6-, and 8-year-old in 2020 and 5-, 7-, and 9- year old in 2021. Results showed that the biomass, yield (seed biomass), soluble sugars, starch, and NSC reserve of Fengdan at the whole tree level increased with the increase in age. Although consistent correlations were observed between soluble sugars, starch and NSC storage, and yield among the plants with different ages, Fengdan showed allometric growth relationships between the accumulation of soluble sugars, starch, and NSC and yield and biomass (standardized major axis analyses slope *b* ≠ 1). Tree age significantly affected biomass and its allocation and NSC levels, especially the yield of Fengdan plants. The results of the investigation of the variations in the relationships between the yield and seasonal fluctuations of NSC and biomass indicate that roots is the key storage structure, whereas stems both serve as sink and/or source functions for the adult (7–9a) plants. NSC level, particularly the concentration of soluble sugars, in stems mainly influences Fengdan yield. These findings together provide new insights into the mechanisms underlying the yield formation of Fengdan and have implications for manipulating sink-source relationship to achieve high yield.

## Introduction

Non-structural carbohydrates (NSC), which mostly consist of soluble sugars and starch, constitutes the primary resource supply (Kozlowski, 1992; Pan et al., 2002). NSC can be used as a temporary carbon source to supply energy for maintaining the normal physiological activities of plants when photosynthesis cannot provide sufficient carbohydrate supply for plant growth and reproduction (Kozlowski, 1992). The variation of NSC is related to the intensity and efficiency of photosynthesis, as well as the development stage, lifeform, and growth environment of plants (Dietze et al, 2014; Furze et al., 2019). Perennial plants usually display periodic dormancy-growth cycle caused by intermittent disturbances of abiotic stresses, such as drought and cold, often faced with the fluctuations in energy supply and demand. Considering the role of NSC on resource supply, the survival of trees over the years depends on their ability to accumulate sufficient NSC in the storage structures, i.e., NSC reserves (Dietze et al, 2014). NSC reserves, especially those present near the end of growth season or dormancy stage, can influence various aspects of tree physiology, including spring growth, flowering, and final yield (Zwieniecki et al, 2022).

NSC reserves in plants have spatial and temporal heterogeneity because of plant age, individual size, organ, and developmental stage. For perennial woody plants, twigs, stems, roots and fruits are all storage structures of NSC, but the concentrations and accumulations of NSC differ among organs. Twigs link the leaves (the carbon source of photosynthates, and stem and root (the main storages of NSC) (Gruber et al., 2013). The variation of NSC in twigs is closely involved in plant growth strategies, thus reflecting the adaptability of plants to the environment changes during key phenological periods (Zhang et al., 2013). The NSC stored in roots and stems supply resources to maintain the respiration of trees during dormant period and to flourish growth and development in the next growth season (Ziemer, 1971; Richardson et al., 2013). According to the principle of nearest allocation of resources, the carbohydrates produced by leaves through photosynthesis is prior to allocated to twigs and fruits. If excessive much NSC is allocated to harvest organs such as fruits, the storage of NSC in roots and stems becomes insufficient, thus impairing the growth and development of plants in the next growth season and thus decreasing the yield; this phenomenon is known as alternate bearing (year-to-year deviation in yield) (Bustan et al, 2011). Therefore, the yield of perennial woody crops is related to the photosynthesis of the current year, as well as the accumulation of NSC in previous years. However, the contribution of NSC to crop yield has not been fully determined (Bustan et al, 2011). The relationship between NSC and yield is complex, thus requiring further understanding of the factors that influence the yield of perennial woody crops.

Fengdan (*Paeonia ostii* T Hong et J X Zhang) is a newly developed oil crop and one of the main cultivation varieties of oil peony in China. This plant is a perennial deciduous shrub, and its light assimilation function is completed by the annual twigs. The process from seed germination, subsequent flowering and fruiting stage, and finally the adult stage takes 3–5 years. During the adult stage of Fengdan, nearly all the annual branches (fresh twigs) set fruits that develop from a single flower on top of a twig. The 3–5 axillaries buds at the bottom of the twigs can survive the winter and develop into new twigs in the next year, forming the new canopy of Fengdan in the coming year (Wang et al., 2022). Since Fengdan was listed as a new woody oil crop by the Chinese government in 2013, several studies have investigated the roles of biomass allocation (Ma et al., 2018), the nutrient elements (Xu et al., 2021), and twig location (Wang et al., 2022) on yield. However, the variations of NSC in Fengdan have not been examined. The variations in NSC, including variations between different organs and the same structure during different phenological period, influence the fruit formation and oil accumulation of Fengdan, thus affecting the yield formation. Carbon supply is a prerequisite for fatty acid synthesis and lipid accumulation. Increasing the supply of photosynthetic metabolites (soluble sugars) during fruit development may enhance the “reservoir strength” of seeds, which can substantially improve the oil content of seeds. After fruit ripening, the photosynthetic metabolites increase the accumulation of NSC in roots and stems, thus providing carbon sources for the development of flower buds and the early growth of the following year. Consequently, it influences the yield. The pattern of NSC variations in Fengdan at both organ and whole tree levels and its contribution to yield were determined using 4- (4a), 6- (6a), and 8-(8a)year old Fengdan population to investigate the variations in biomass, soluble sugars, starch, and NSC during the dormant and fruit ripening period. The following questions were answered: 1) What is the dynamics of NSC and how it is affected by tree age? 2) Is there a significant contribution of NSC to the yield of Fengdan?

## Materials and methods

The experiments were conducted in a commercial oil Peony orchard (31°26’N, 120°36’E, ~30 m above sea level) located in Suzhou City, Jiangsu Province, China. This orchard included Fengdan populations aged 2–10 years, and each population covered an area of ~600 m^2^. In the orchard, the adult Fengdan plants (flowering and fruiting, usually ≥3 years old) were individually cultivated at 1 m × 1 m spacing, and all populations were applied with the same water-fertilizer management.

The effect of tree age was determined by selecting 4a, 6a, and 8a Fengdan populations as experimental populations, and samplings were carried out for two consecutive years (2020–2021) for each population on two time points in the dormancy and fruit ripening periods (January 25 and August 5), which are the two key stages for the survival or growth and reproduction of Fengdan plants (Li et al., 2011). On each sampling occasion, five individuals of Fengdan in each aged population with similar plants size (height and number of branches) were randomly selected and excavated up to 40–50 cm from their rooting point. The excavated plants were washed with water to remove the soil and brought back to the laboratory. In the laboratory, the plants were separated into the roots, stems (including main stems and branches older than 1-year-old), twigs (annual branches, almost all set fruits (Wang et al., 2022)), leaves, and fruits (shells and seeds). All plant materials were vigorously brushed to remove soil particles, cut in small pieces, and oven-dried to a constant weight at 65°C. The dry weight of samples was measured to determine the root biomass, stem biomass, twig biomass, leaf biomass, fruit biomass, including seed and fruit husk biomass, and whole tree biomass. The sum of the biomass of all organs was calculated as whole tree biomass = root biomass+ stem biomass + twig biomass + leaf biomass + seed biomass + husk biomass.

The soluble sugar content in each organ was determined using the anthrone colorimetric method (Gary & Klausmeier, 2002), and starch content was determined using the dual-wavelength method (Zhu et al., 2008). NSCs mostly consists of soluble sugars and starches; therefore, the NSC content is roughly equal to the sum of soluble sugar and starch contents (Audrey et al., 2015). The accumulation of soluble sugars, starch, and NSC in each organ and whole tree was obtained by multiplying the biomass with the corresponding contents. The percentages of biomass, soluble sugars, starch, and NSC were obtained by dividing the quantity of each organ by the quantity of the whole plant ×100.

In the present study, the three aged populations (4a, 6a, and 8a) were sampled firstly in 2020 and again in 2021, resulting in six aged populations (2020: 4a, 6a, 8a; 2021: 5a, 7a, 9a) were included. The first flowering date of the sampled Fengdan populations in 2020 is 2 days earlier than that in 2021, indicating that the climate change in the two years is small, and the influence of the inter-annual climate difference on Fengdan physiology is negligible. Thus, the samples in the two consecutive years were used as that with consecutive ages (4a–9a) for the analysis. Where necessary, the measured variables were logarithmically transformed (based on 10) to meet the assumptions of normality and homogeneity of variance. One-way ANOVA was used to test the effects of age on the variables. Then, the differences in variables among the plants with different ages were determined using the least significant difference method. Paired *t*-test was used to check whether the contents and accumulation of soluble sugars, starch, and NSC in the same organ were different between the dormant and fruit ripening periods. These analyses were performed using the software package IBM SPSS version 22.0 for Windows (SPSS Inc., IBM Company Chicago, IL, USA, 2010). The allometric growth equation *y* = *aX^b^* was used to describe the relationship between NSC content in the roots, stems, twigs, and seed mass of Fengdan plants. After logarithmic transformation, the equation can be expressed as log (*y*) = log (*a*) + *b*log (*x*), where *x* and *y* are NSC accumulation and biomass, respectively. Parameter *a* is usually referred to as the “allometric coefficient”, and *b* is the “allometric exponent.” An exponent that is significantly different from 1.0 indicates an allometric (non-isometric) relationship. Standardized major axis (SMA) was used to estimate the parameters and test whether the slope of each population or subpopulation statistically differ from one. All SMA analyses were conducted using the software package “Standardised Major Axis Tests and Routines (SMATR)” (Falster et al., 2006). The significance level for testing slope heterogeneity and difference from slope = 1 was *P*<0.05.

## Results

### Biomass dynamics

**Figure 1:**
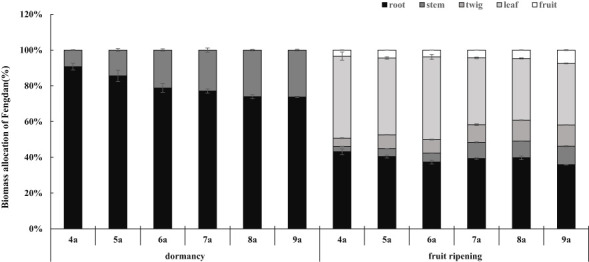
Biomass allocation of Fengdan.

ANOVA analysis showed that the tree age significantly impacted the biomass of Fengdan (**Table 1**). Especially, total biomass either during the dormancy period or the fruit ripening period and the yield per plant (seed biomass) significantly increased with the increase in tree age (**Table 2**). During the dormancy period, the biomass was mostly allocated to the roots, and the percentage of biomass in roots decreased with the increase in tree age, whereas a reversal tendency occurred in stems (**Figure 1**). **Figure 1** also shows that the biomass of Fengdan plant was mostly allocated to the roots and stems during the fruit ripening period, and the percentage of biomass in twigs and fruits increased with the increase in tree age.

**Table 1:** *F*-values of One-way ANOVA for the effects of tree age (df=5) on the biomass, biomass allocation, the concentrations and accumulations of soluble sugar, starch, and NSC in each organ and whole-plant of Fengdan during dormancy/fruit ripening.

Variable	Whole-plant	Root	Stem	Twig	Leaf	Fruit	Seed
Biomass	1806.53^**^/1880.04^**^	1215.42^**^/160.698^**^	1073.909^**^/105.395^**^	–/508.961^**^	–/26.213^**^	–/55.90^**^	–/1362.27^**^
Biomass allocation	–	29.320**/17.000**	235.925**/643.975**	–/282.056**	–/55.897**	–/320.070**	–/87.264**
Soluble sugar concentration	21.380**/11.571**	9.942^**^/33.691^**^	21.366^**^/28.404^**^	–/0.285	–/1.019	–/27.611^**^	–/22.079**
Soluble sugar accumulation	79.821**/375.714**	44.106^**^/310.459^**^	30.805^**^/71.408^**^	–/456.97^**^	–/32.778^**^	–/260.47^**^	–/229.204**
Starch concentration	53.373**/27.353**	37.634^**^/9.540^**^	10.179^**^/13.642^**^	–/13.642^**^	–/4.955^**^	–/18.349^*^	–/120.043**
Starch accumulation	31.843**/230.839**	18.695^**^/72.524^**^	233.83^**^/95.334^**^	–/126.12^**^	–/8.188^**^	–/254.64^**^	–/269.295**
NSC concentration	56.042**/36.166**	36.43^**^/20.410^**^	6.681^**^/0.952	–/8.577^**^	–/2.555	–/19.489^**^	–/87.350**
NSC accumulation	62.541**/368.777**	36.135^**^/126.954^**^	76.054^**^/126.348^**^	–/524.293^**^	–/26.213^**^	–/286.952^**^	–/288.190**

^*^ *P* < 0.05; ^**^ *P* < 0.01.

**Table 2:** Average values of biomass (± SD) (g) of each organ and whole-plant of Fengdan during dormancy/fruit ripening.

Tree age	Whole-plant	Root	Stem	Twig	Leaf	Fruit	Seed
4a	106.03 ± 1.93^f^/215.24 ± 10.1^e^	96.18 ± 1.97^f^/92.88 ± 3.33^b^	9.85 ± 0.25^f^/6.44 ± 0.32^d^	–/9.90 ± 0.50^d^	–/98.75 ± 4.94^d^	–/7.27 ± 0.31^e^	–/4.17 ± 0.43^e^
5a	148.22 ± 6.12^e^/296.70 ± 2.26^d^	126.85 ± 4.82^e^/119.88 ± 2.25^b^	21.37 ± 1.23^e^/13.29 ± 0.24^c^	–/22.92 ± 0.42^c^	–/127.47 ± 1.86^c^	–/13.13 ± 0.23^d^	–/7.30 ± 0.08^d^
6a	211.01 ± 7.38^d^/345.7 ± 6.3^c^	166.19 ± 5.20^d^/129.02 ± 3.72^b^	44.82 ± 1.40^d^/17.37 ± 0.54^c^	–/26.10 ± 0.77^c^	–/160.03 ± 4.55^bc^	–/13.24 ± 0.22^d^	–/8.88 ± 0.53^c^
7a	318.67 ± 3.43^c^/495.32 ± 3.60^b^	245.58 ± 3.76^c^/194.65 ± 2.45^ab^	73.09 ± 3.62^c^/44.93 ± 1.2^b^	–/49.01 ± 2.30^b^	–/185.15 ± 2.15^b^	–/21.58 ± 0.36^c^	–/14.47 ± 0.86^b^
8a	372.47 ± 6.08^b^/560.61 ± 8.89^a^	275.49 ± 4.01^b^/222.73 ± 5.74^a^	96.98 ± 1.43^b^/51.92 ± 0.94^b^	–/66.09 ± 1.18^ab^	–/193.60 ± 1.72^ab^	–/26.27 ± 0.85^b^	–/14.85 ± 0.52^b^
9a	417.78 ± 3.75^a^/617.55 ± 1.86^a^	307.97 ± 1.57^a^/221.27 ± 1.26^a^	109.81 ± 1.85^a^/64.22 ± 1.64^a^	–/73.17 ± 0.94^a^	–/212.65 ± 1.49^a^	–/46.24 ± 1.49^a^	–/26.14 ± 0.92^a^

Different lowercase letters denote significant differences between ages according to the least significant difference test at P = 0.05.

### Variations of soluble sugars, starch and NSC

**Figure 2:**
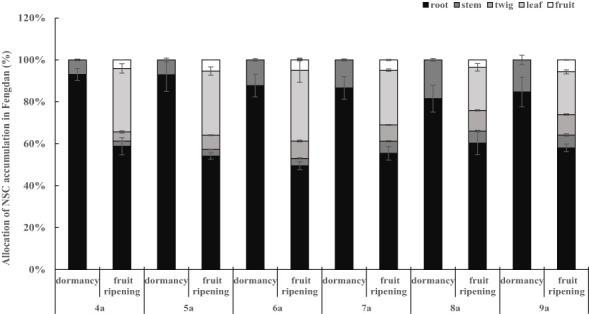
Allocation of NSC in Fengdan.

The concentrations and accumulations of soluble sugars (except for that in twigs and leaves during the fruit ripening period), starch, and NSC (except for that in stems and leaves during the fruit ripening period) in the organs of Fengdan were significantly influenced by tree age (ANOVA, *P*<0.05; **Table 1**). The concentrations of soluble sugars in roots during dormancy, in stems either during the dormancy or fruit ripening periods and in fruits decreased with the increase in tree age, while the opposite was observed in the roots during the fruit ripening period (**Tables 3**, **4**). **Tables 3** and **4** show that starch concentrations differed between organs, and that in the stems and roots during the fruit ripening period and in the stems during dormancy increased with tree age. The concentrations of NSC in the roots and stems during dormancy and in the stems during the fruit ripening period decreased with tree age, whereas an opposite trend was observed in the roots during the fruit ripening period (**Tables 3**, **4**). The accumulations of soluble sugars, starch, and NSC in all organs increased with tree age (**Tables 3**, **4**). The allocation of NSC to twigs and leaves decreased with the increase in tree age (**Figure 2**).

**Table 3:** Average values of the concentrations and accumulations soluble sugar, starch, and NSC of each organ and whole-plant of Fengdan during dormancy.

	Tree age	Root	Stem	
**Soluble sugar concentration** **(%)**	4a	16.10% ± 0.50%^a^	15.14% ± 0.56%^a^	
	5a	14.68% ± 0.52%^a^	11.41% ± 0.81%^b^	
	6a	12.87% ± 0.66%^b^	10.25% ± 0.98%^bc^	
	7a	12.33% ± 1.22%^b^	9.43% ± 0.46%^c^	
	8a	11.86% ± 0.53%^b^	8.64% ± 0.73%^cd^	
	9a	12.33% ± 1.22%^b^	6.98% ± 1.61%^d^	
**Starch concentration** **(%)**	4a	25.37% ± 1.29%^a^	4.17% ± 0.98%^c^	
	5a	20.49% ± 1.22%^b^	4.36% ± 0.42%^c^	
	6a	15.28% ± 1.25%^c^	4.36% ± 0.42%^c^	
	7a	14.68% ± 1.53%^c^	4.55% ± 0.48%^c^	
	8a	10.40% ± 1.36%^d^	5.66% ± 0.03%^b^	
	9a	15.28% ± 1.25%^c^	6.98% ± 0.40%^a^	
**NSC concentration** **(%)**	4a	41.47% ± 1.98%^a^	19.31% ± 1.28%^a^	
	5a	35.17% ± 1.95%^b^	15.67% ± 1.38%^b^	
	6a	28.15% ± 1.12%^c^	14.61% ± 1.38%^b^	
	7a	27.02% ± 2.02%^c^	13.99% ± 0.28%^b^	
	8a	22.25% ± 1.74%^d^	14.30% ± 0.79%^b^	
	9a	27.62% ± 2.69%^c^	13.95% ± 2.25%^b^	
**Soluble sugar accumulation (g)**	4a	15.48 ± 0.38^c^	1.60 ± 1.49^c^	
	5a	18.64 ± 1.33^cd^	2.44 ± 0.31^c^	
	6a	21.37 ± 0.68^c^	4.58 ± 0.30^b^	
	7a	30.25 ± 2.54^b^	6.88 ± 0.21^a^	
	8a	32.68 ± 1.92^b^	8.38 ± 0.63^a^	
	9a	37.97 ± 3.62^a^	8.66 ± 1.78^a^	
**Starch accumulation (g)**	4a	24.38 ± 0.88^c^	0.41 ± 0.10^e^	
	5a	26.03 ± 2.49^c^	0.93 ± 0.14^e^	
	6a	25.45 ± 2.89^c^	1.95 ± 0.17^d^	
	7a	28.66 ± 4.00^c^	3.33 ± 0.45^c^	
	8a	38.66 ± 3.92^b^	5.49 ± 0.10^b^	
	9a	47.05 ± 3.61^a^	7.66 ± 0.46^a^	
**NSC accumulation (g)**	4a	39.86 ± 1.33^b^	1.90 ± 0.17^d^	
	5a	44.68 ± 4.27^b^	3.38 ± 0.51^d^	
	6a	46.82 ± 3.25^b^	6.53 ± 0.42^c^	
	7a	66.33 ± 4.64^a^	10.21 ± 0.45^b^	
	8a	65.05 ± 6.64^a^	14.21 ± 2.00^a^	
	9a	67.16 ± 8.06^a^	15.32 ± 2.48^a^	

Different lowercase letters denote significant differences between ages according to the least significant difference test at P = 0.05.

**Table 4:** Average values of the concentrations and accumulations soluble sugar, starch, and NSC of each organ and whole-plant of Fengdan during fruit ripening.

	Tree age	Root	Stem		Twig	Leaf	Fruit
**Soluble sugar concentration** **(%)**	4a	11.21% ± 1.20%^c^	13.87% ± 0.65%^a^		16.02% ± 0.51%	12.11% ± 0.94%	12.52% ± 0.19%^a^
	5a	11.53% ± 0.31%^c^	11.29% ± 0.78%^b^		15.84% ± 0.54%	13.62% ± 0.93%	12.12% ± 0.14%^ab^
	6a	11.48% ± 0.10%^c^	10.91% ± 1.04%^bc^		15.97% ± 0.24%	13.60% ± 1.99%	11.98% ± 1.13%^ab^
	7a	15.43% ± 0.84%^bc^	9.61% ± 0.97%^c^		15.62% ± 0.71%	13.70% ± 0.10%	11.33% ± 0.52%^b^
	8a	16.53% ± 0.94%^ab^	9.40% ± 0.73%^c^		15.97% ± 0.23%	13.65% ± 0.17%	8.59% ± 0.40%^c^
	9a	17.47% ± 0.58%^a^	5.76% ± 0.17%^d^		15.62% ± 0.71%	13.78% ± 0.15%	8.41% ± 0.08%^c^
**Starch concentration** **(%)**	4a	19.63% ± 2.12%^b^	4.97% ± 0.74%^d^		5.40% ± 1.03%^d^	2.86% ± 0.32%^b^	14.64% ± 0.48%^bc^
	5a	20.13% ± 0.08%^b^	5.40% ± 1.03%^d^		4.97% ± 0.74%^d^	3.22% ± 0.35%^b^	16.19% ± 0.55%^b^
	6a	21.00% ± 0.69%^b^	6.21% ± 1.01%^cd^		10.88% ± 1.43%^a^	4.21% ± 0.59%^ab^	19.20% ± 2.02%^a^
	7a	23.41% ± 1.48%^b^	7.66% ± 0.46%^bc^		6.21% ± 1.01%^cd^	5.51% ± 0.20%^a^	19.44% ± 1.14%^a^
	8a	27.46% ± 3.59%^a^	8.27% ± 0.44%^b^		8.27% ± 0.44%^b^	3.54% ± 1.29%^b^	13.25% ± 0.67%^c^
	9a	28.18% ± 1.03%^a^	10.88% ± 1.43%^a^		7.66% ± 0.46%^bc^	2.86% ± 0.87%^b^	12.97% ± 0.07%^c^
**NSC concentration** **(%)**	4a	30.84 ± 1.13%^c^	18.84% ± 0.30%		21.42% ± 1.52%^c^	14.97% ± 0.93%^b^	27.16% ± 0.63%^b^
	5a	31.67% ± 0.35%^c^	16.68% ± 1.28%		20.81% ± 0.89%^c^	16.84% ± 0.88%^ab^	28.31% ± 0.62%^ab^
	6a	32.48% ± 0.79%^c^	17.12% ± 2.03%		26.85% ± 1.27%^a^	17.81% ± 2.57%^ab^	31.18% ± 3.07%^a^
	7a	38.84% ± 2.32%^b^	17.27% ± 1.00%		21.83% ± 1.68%^bc^	19.21% ± 0.29%^a^	30.77% ± 1.45%^a^
	8a	43.99% ± 4.49%^a^	17.67% ± 1.07%		24.23% ± 0.65%^b^	17.19% ± 1.46%^ab^	21.83% ± 1.07%^c^
	9a	45.65% ± 1.60%^a^	16.64% ± 1.44%		23.28% ± 0.68%^bc^	16.64% ± 0.76%^ab^	21.39% ± 0.15%^c^
**Soluble sugar accumulation (g)**	4a	10.38 ± 0.74^d^	0.89 ± 0.06^c^		1.59 ± 0.13^e^	11.97 ± 1.19^e^	0.91 ± 0.03^d^
	5a	13.83 ± 0.58^c^	1.50 ± 0.10^b^		3.63 ± 0.07^d^	17.37 ± 1.38^d^	1.59 ± 0.04^c^
	6a	14.82 ± 0.50^c^	1.89 ± 0.13^b^		4.17 ± 0.09^d^	21.84 ± 3.80^c^	1.58 ± 0.13^c^
	7a	30.03 ± 1.59^b^	4.33 ± 0.52^a^		7.64 ± 0.03^c^	25.36 ± 0.40^b^	2.45 ± 0.15^b^
	8a	36.79 ± 1.49^a^	4.89 ± 0.46^a^		10.55 ± 0.19^b^	26.42 ± 0.45^ab^	2.25 ± 0.05^b^
	9a	38.65 ± 1.19^a^	4.70 ± 0.20^a^		11.43 ± 0.66^a^	29.29 ± 0.16^a^	3.89 ± 0.13^a^
**Starch accumulation (g)**	4a	18.29 ± 2.61^d^	0.32 ± 0.06^d^		0.54 ± 0.13^d^	2.81 ± 0.17^c^	1.06 ± 0.01^d^
	5a	24.14 ± 0.55^cd^	0.72 ± 0.12^d^		1.14 ± 0.16^c^	4.11 ± 0.48^b^	2.13 ± 0.06^c^
	6a	27.09 ± 1.20^c^	1.07 ± 0.14^d^		2.84 ± 0.38^b^	6.76 ± 1.11^a^	2.54 ± 0.24^c^
	7a	45.57 ± 2.82^b^	3.44 ± 0.13^d^		3.03 ± 0.37^b^	6.20 ± 0.43^a^	4.19 ± 0.26^b^
	8a	61.07 ± 7.30^a^	4.29 ± 0.25^b^		5.47 ± 0.32^a^	6.86 ± 2.51^a^	3.48 ± 0.09^b^
	9a	62.34 ± 2.06^a^	6.99 ± 0.95^a^		5.60 ± 0.29^a^	6.09 ± 1.87^a^	6.00 ± 0.18^a^
**NSC accumulation (g)**	4a	32.14 ± 1.91^d^	1.06 ± 0.12^e^		2.13 ± 0.28^f^	14.78 ± 1.22^d^	1.97 ± 0.04^f^
	5a	41.88 ± 1.37^c^	2.66 ± 0.08^d^		4.77 ± 0.15^e^	21.48 ± 1.58^c^	3.72 ± 0.09^e^
	6a	46.29 ± 1.93^c^	2.96 ± 0.31^d^		7.09 ± 0.45^d^	28.60 ± 5.47^b^	4.12 ± 0.40^d^
	7a	75.60 ± 4.93^b^	7.45 ± 0.99^c^		10.67 ± 0.41^c^	35.56 ± 0.91^a^	6.64 ± 0.41^c^
	8a	88.95 ± 5.44^a^	11.47 ± 0.97^a^		16.02 ± 0.57^b^	33.28 ± 3.26^ab^	5.73 ± 0.16^b^
	9a	90.45 ± 3.14^a^	8.02 ± 1.14^b^		17.03 ± 0.71^a^	35.38 ± 1.91^a^	9.89 ± 0.34^a^

Different lowercase letters denote significant differences between ages according to the least significant difference test at P = 0.05.

Paired *t*-test revealed that the concentrations and accumulations of soluble sugars, starch, and NSC in the roots and stems of each aged Fengdan significantly differed between the dormancy and fruit ripening period (**Table 5**). From the dormancy stage to the fruit ripening stage, the accumulations of soluble sugars in the roots of 4a–6a Fengdan and in the stems of 4a–5a plants decreased, whereas those in the 7a–9a roots and 6a–9a stems increased. The accumulations of starch increased in the 4a–5a roots and decreased in the roots of 6a–9a plants, whereas those in the stems of each aged plants increased. The accumulation of NSC in the roots and stems decreased, and more drastic variations in NSC accumulation occurred in stems compared with that in the roots (**Table 5**).

**Table 5:** Concentration and accumulation of soluble sugar, starch, and NSC in each organ of Fengdan during dormancy and fruit ripening.

	Age	Organ	Dormancy	Fruit ripening	*P* value of paired-tiests
**Soluble sugar concentration**	4a	root	16.10% ± 0.50%	11.21% ± 1.20%	0.004
		stem	15.14% ± 0.56%	13.87% ± 0.65%	0.001
	5a	root	14.68% ± 0.52%	11.53% ± 0.31%	0.001
		stem	11.41% ± 0.81%	11.29% ± 0.78%	0.011
	6a	root	12.87% ± 0.66%	11.48% ± 0.10%	0.032
		stem	10.25% ± 0.98%	10.91% ± 1.04%	0.000
	7a	root	12.33% ± 1.22%	15.43% ± 0.84%	0.032
		stem	9.43% ± 0.46%	9.61% ± 0.97%	0.002
	8a	root	11.86% ± 0.53%	16.53% ± .94%	0.003
		stem	8.64% ± 0.73%	9.40% ± 0.73%	0.002
	9a	root	12.33% ± 1.22%	17.47% ± 0.58%	0.004
		stem	6.98% ± 1.61%	5.76% ± 0.17%	0.027
**Starch concentration**	4a	root	25.37% ± 1.29%	19.63% ± 2.12%	0.023
		stem	4.17% ± 0.98%	4.97% ± 0.74%	0.371
	5a	root	20.49% ± 1.22%	20.13% ± 0.08%	0.035
		stem	4.36% ± 0.42%	5.40% ± 1.03%	0.221
	6a	root	15.28% ± 1.25%	21.00% ± 0.69%	0.003
		stem	4.36% ± 0.42%	6.21% ± 1.01%	0.005
	7a	root	14.68% ± 1.53%	23.41% ± 1.48%	0.003
		stem	4.55% ± 0.48%	7.66% ± 0.46%	0.002
	8a	root	10.40% ± 1.36%	27.46% ± 3.59%	0.002
		stem	5.66% ± 0.03%	8.27% ± 0.44%	0.001
	9a	root	15.28% ± 1.25%	28.18% ± 1.03%	0.000
		stem	6.98% ± 0.40%	10.88% ± 1.43%	0.015
**NSC concentration**	4a	root	41.47% ± 1.77%	30.84% ± 1.13%	0.001
		stem	19.31% ± 1.14%	18.84% ± 0.30%	0.000
	5a	root	35.17% ± 1.74%	31.67% ± 0.35%	0.038
		stem	15.76% ± 1.24%	16.68% ± 1.28%	0.035
	6a	root	28.15% ± 1.01%	32.48±%0.79%	0.006
		stem	14.61% ± 1.24%	17.12% ± 2.03%	0.005
	7a	root	27.02% ± 1.81%	38.84% ± 2.32%	0.003
		stem	13.99% ± 0.25%	17.27% ± 1.00%	0.008
	8a	root	22.25% ± 1.55%	43.99% ± 4.49%	0.002
		stem	14.30% ± 0.70%	17.67% ± 1.07%	0.015
	9a	root	27.62% ± 2.40%	45.65% ± 1.60%	0.001
		stem	13.95% ± 2.01%	16.64% ± 1.44%	0.004
**Soluble sugar accumulation**	4a	root	15.48 ± 0.38	10.38 ± 0.74	0.001
		stem	1.49 ± 0.09	0.89 ± 0.06	0.027
	5a	root	18.64 ± 1.33	13.83 ± 0.58	0.011
		stem	2.44 ± 0.31	1.50 ± 0.10	0.003
	6a	root	21.37± 0.68	14.82 ± 0.50	0.000
		stem	4.58 ± 0.30	1.89± 0.13	0.005
	7a	root	30.25 ± 2.54	30.03 ± 1.59	0.002
		stem	6.88 ± 0.21	4.33 ± 0.52	0.001
	8a	root	32.68 ± 1.92	36.79 ± 1.49	0.002
		stem	8.38 ± 0.63	4.89 ± 0.46	0.004
	9a	root	37.97 ± 3.62	38.65 ± 1.19	0.027
		stem	7.66 ± 1.78	3.70 ± 0.20	0.000
**Starch accumulation**	4a	root	24.38 ± 0.88	18.29 ± 2.61	0.002
		stem	0.41 ± 0.10	0.32 ± 0.06	0.000
	5a	root	26.03 ± 2.49	24.14 ± 0.55	0.005
		stem	0.93 ± 0.14	0.72 ± 0.12	0.000
	6a	root	25.45 ± 2.89	27.09 ± 1.20	0.035
		stem	1.95 ± 0.17	1.07 ± 0.14	0.005
	7a	root	36.08 ± 4.00	45.57 ± 2.82	0.000
		stem	3.33 ± 0.45	6.44 ± 0.13	0.000
	8a	root	28.66 ± 3.92	61.07 ± 7.30	0.000
		stem	5.49 ± 0.10	4.29 ± 0.25	0.000
	9a	root	47.05 ± 3.61	62.34 ± 2.06	0.000
		stem	7.66 ± 0.46	6.99 ± 0.95	0.001
**NSC accumulation**	4a	root	39.86 ± 1.19	28.67 ± 2.00	0.002
		stem	1.90 ± 0.15	1.21 ± 0.08	0.002
	5a	root	44.68 ± 3.82	37.97 ± 1.09	0.000
		stem	3.38 ± 0.45	2.21 ± 0.13	0.001
	6a	root	46.82 ± 2.90	41.91 ± 1.64	0.000
		stem	6.53 ± 0.38	2.96 ± 0.26	0.001
	7a	root	66.33 ± 4.15	75.60 ± 4.41	0.000
		stem	10.22 ± 0.40	7.76 ± 0.53	0.003
	8a	root	61.34 ± 4.87	97.86 ± 8.78	0.000
		stem	13.86 ± 0.55	9.18 ± 0.67	0.005
	9a	root	85.02 ± 7.03	101.00 ± 3.23	0.001
		stem	15.32 ± 2.22	10.69 ± 1.01	0.000

### Relationship between NSC and biomass and yield

An allometric relationship was observed between the individual yield and total soluble sugars accumulations of 4a, 6a–9a Fengdan, between the yield and total starch accumulations of 5a–9a plants, and between the yield and total NSC accumulation of 4a, 6a, 8a, and 9a plants (*P*<0.05) (**Table 6**). An allometric relationship was also observed between the total biomass and total soluble sugars storage of 5a, 6a, and 9a plants and total starch storage of 6a and 9a Fengdan. It was also observed and between the total biomass and total NSC storage of 5a, 6a, and 9a plants (*P*<0.05, **Table 6**).

**Table 6:** The allometric growth relationship between seed biomass and total biomass and NSC accumulation of each organ and whole-plant of Fengdan during fruit ripening.

Parameter	Tree age	Allometric indexes			Allometric constants
		*R^2^*	*P value*	Slope(95% *CI*)	Intercept(95% *CI*)
Y = seed biomass X = total soluble sugar accumulation	4a	0.950	0.001	-3.79(-5.149~-2.79)	5.967(4.306~7.628)
	5a	0.431	0.157	0.3394(0.2754~0.4183)	0.7079 0.6752~0.7406
	6a	0.695	0.039	0.523(0.1336~0.687)	0.7972(0.6550~0.9395)
	7a	0.996	0.000	0.460(0.419~0.504)	0.325(0.251~0.399)
	8a	0.994	0.003	-3.65(-5.493~-2.425)	8.134(5.208~11.061)
	9a	0.804	0.016	-6.697(-11.983~-3.743)	1.6882(1.5400~1.8364)
Y = seed biomass X = total starch accumulation	4a	0.385	0.189	-0.7946(-1.9076~-0.331)	0.8764(0.6118~1.1410)
	5a	0.844	0.010	-0.815(-1.376~-0.483)	1.064(0.912~1.215)
	6a	0.958	0.001	1.102(0.832~1.460)	-0.822(-1.326~2.191)
	7a	0.801	0.016	-8.149(-14.635~-4.538)	15.96(6.76~25.15)
	8a	0.994	0.003	-0.552(-0.763~-0.399)	8.134(5.208~11.061)
	9a	0.804	0.015	2.436(1.362~4.357)	2.432(1.111~3.753)
Y = seed biomass X = total NSC accumulation	4a	0.560	0.087	-1.509(-1.2692~-1.218)	3.165(0.828~5.502)
	5a	0.517	0.107	-0.0931(-0.1126~-0.077)	0.9361(0.9222~0.9501)
	6a	0.789	0.018	0.704(0.1798~0.5209)	0.4944(0.2394~0.7494)
	7a	0.399	0.179	-1.247(-3.1754~-0.4899)	2.6324(1.0470~4.2177)
	8a	0.970	0.010	-0.979(-1.2394~-0.773)	0.8078(0.6077~1.0078)
	9a	0.9806	0.015	7.526(4.221~13.418)	0.8353(0.6239~1.0467)
Y = total biomass X = total soluble sugar accumulation	4a	0.285	0.275	1.447(0.533~3.391)	0.291(-2.101~2.684)
	5a	0.965	0.000	0.218(0.168~0.282)	2.238(2.043~2.227)
	6a	0.989	0.000	0.322(0.279~0.372)	2.007(1.935~2.091)
	7a	0.665	0.048	-10.35(-21.60~-4.986)	19.95(4.81~35.08)
	8a	0.013	0.830	2.374(0.770~7.318)	-1.782(-8.028~4.464)
	9a	1.000	0.000	-0.507(-0.510~-0.503)	3.776(3.769~3.783)
Y = total biomass X = total starch accumulation	4a	0.290	0.270	-0.682(0.252~-1.847)	1.363(0.233~2.493)
	5a	0.146	0.454	1.312(0.451~3.817)	0.426(-2.200~3.050)
	6a	0.991	0.000	0.524(0.461~0.596)	1.695(1.585~1.804)
	7a	0.188	0.391	1.310(0.459~3.736)	0.402(-2.467~3.271)
	8a	0.445	0.148	0.287(0.116~0.0.709)	2.235(1.705~2.765)
	9a	0.999	0.000	0.037(0.034~0.037)	2.728(2.725~2.730)
Y = total biomass X = total NSC accumulation	4a	0.758	0.024	1.463(0.773~2.768)	-0.177(-1.89~1.53)
	5a	0.998	0.000	0.407(0.381~0.434)	1.711(1.662~1.761)
	6a	0.990	0.000	0.394(0.344~0.452)	1.775(1.670~1.880)
	7a	0.111	0.519	0.778(0.263~2.300)	1.060(-1.08~3.201)
	8a	0.563	0.086	0.728(0.320~1.657)	1.180(-0.259~2.618)
	9a	1.000	0.000	0.099(0.097~0.101)	2.576(2.573~2.580)

The yield per plant of the young Feangdan (4–6a) was negatively correlated with the concentrations of soluble sugars in the roots and stems during dormancy, in the stems during fruit ripening, with the concentrations of starch in the roots during dormancy, with that of NSC in the roots and stems during dormancy, and in the stems during fruit ripening; while it was positively correlated with the concentrations of soluble sugars in the roots during fruit ripening, with that of NCS in the roots during fruit ripening, and with the accumulations of soluble sugars, starch and NSC of each organ and whole-plant during both periods (**Table 7**). **Table 7** showed that, for the adult Fengdan (7–9a), the yield was negatively correlated with the concentrations of soluble sugars in the stem during dormancy and fruit ripening and with the accumulations of soluble sugars during fruit ripening and starch during dormancy in the stems. A significant positively correlation was observed between the yield of adult Fengdan and the concentrations of soluble sugars in the roots during fruit ripening and starch in the stems during dormancy and fruit ripening; the accumulations of soluble sugars in the roots and whole plant during dormancy and fruit ripening and in the twigs and leaves; and the accumulations of starch and NSC in the roots during dormancy, the stems during fruit ripening and the whole plants during dormancy and fruit ripening (**Table 7**). Regardless of age effect, the yield per plant is positively correlated with the accumulations of soluble sugars, starch, and NSC in each organ and whole-plant and with the concentrations of soluble sugars, starch, and NSC in the roots during fruit ripening and that of starch in the stems during dormancy and fruit ripening; whereas a significantly negative correlation between the yield and the concentrations of soluble sugars, starch, and NSC in the roots during fruit ripening and that of soluble sugars and starch in the stems during dormancy and fruit ripening (**Table 7**).

**Table 7:** The *r* coefficients of pearson correlations between individual yield (seed biomass) and the concentrations and accumulations of soluble sugar, starch, and NSC in each organ of Fengdan during dormancy/fruit ripening.

Tree age	Organ	Concentration			Accumulation		
		Soluble sugar	Starch	NSC	Soluble sugar	Starch	NSC
4-6a	root	-0.904**/0.667**	-0.938**/0.438	-0.939**/0.807**	0.950**/0.988**	0.301/0.932**	0.803**/0.977**
	stem	-0.934**/-0.908**	0.145/0.483	-0.884**/-0.625*	0.901**/0.951**	0.920**/0.917**	0.944**/0.946**
	twig	–/0.099	–/0.655*	–/0.628*	–/0.981**	–/0.861**	–/0.971**
	leaf	–/0.539	–/0.814**	–/0.707*	–/0.908**	–/0.900**	–/0.918**
	whole plant	–	–	–	0.946**/0.972**	0.540/0.957**	0.894**/0.972**
7-9a	root	0.104/0.654*	0.519/0.447	0.474/0.512	0.777*/0.645*	0.843**/0.501	0.893**/0.551
	stem	-0.641**/-0.932**	0.865**/0.856**	0.060/-0.376	0.025/-0.690*	-0.902**/0.919**	0.621*/0.782**
	wig	–/-0.219	–/0.214	–/0.082	–/0.677*	–/0.553	–/0.633*
	leaf	–/0.382	–/-0.566	–/-0.525	–/0.951**	–/-0.472	–/0.275
	whole plant	–	–	–	0.710**/0.719**	0.796**/0.614*	0.931**/0.665*
All	root	-0.672**/0.894**	-0.594**/0.818**	-0.626**/0.871**	0.932**/0.899**	0.903**/0.880**	0.963**/0.889**
	stem	-0.827**/-0.947**	0.861**/0.924**	-0.591**/-0.329	0.783**/0.708**	0.046/0.969**	0.900**/0.917**
	twig	–/-0.295	–/0.216	–/0.146	–/0.914**	–/0.865**	–/0.907**
	leaf	–/0.370	–/0.009	–/0.222	–/0.873**	–/0.426*	–/0.796**
	whole plant	–	–	–	0.913**/0.904**	0.926**/0.898**	0.972**/0.902**

^*^ *P* < 0.05; ^**^ *P* < 0.01.

## Discussion

### Biomass dynamics of Fengdan

Our findings confirm that tree age significantly affects the biomass allocation of woody plants (Jagodzinski et al., 2016). Fengdan biomass and its partitioning among different organs changed with tree ages, which is consistent with the findings of Wang et al. (2017). The biomass of individual twig did not differ between different aged Fengdan plants (Ma et al., 2018). But, the total twig biomass per Fengdan plant increased with tree age increasing (**Table 2**), suggesting that the number of twigs mainly contributes to the age effects on the increase of biomass and yield, as well as the yield of Fengdan.

Seasonal variations in biomass allocation reflect the capacity of energy accumulation of plant through photosynthesis and the adjustment of source-sink structure within plant at different stages of life history (Sadras & Denison, 2009; Kudo & Ida, 2010). As a sub shrub, Fengdan has alternating growth and dormancy in its annual cycle. Based on 2 years of investigation, the results showed significant biomass changes of Fengdan plants between the dormancy (the period has the largest reserve and slowest metabolism) and fruit ripening (the period of sink dominance of fruit over reserve formation) stages. For all age Fengdan plants, the biomass partitioning of both roots and stems decreased from dormancy to fruit ripening stages (**Figure 1**). Such pattern suggests that besides being sink structures, they provided nutrients for bud sprouting and the development of young leaves and flowers at the early spring growth stage, thus acting as source structures. In addition, according to the principle of assimilate proximate distribution, the stems play key roles in providing and transporting nutrients for the recovery of growth from dormancy, thus acting as the key source structures at the beginning of growth (Wang et al., 2018).

### NSC fluctuations in Fengdan

NSC bridges the source-sink relationship within crops. It buffers the damage caused by stress through osmotic regulation and is an important source of carbon required for grain filling and bud sprouting (Raessler et al., 2010). The concentrations of NSC may dynamically be modulated among different organs depending on the growth strategies of plants (Mooney & Hays, 1973). The concentrations of NSC and soluble sugars in the roots and stems of Fengdan decreased with the increase of tree ages during dormancy but not in the fruit ripening stage. These results were obtained, possibly because the younger Fengdan plants are relatively sensitive to low-temperature stress compared with the older ones. Therefore, the former needs much higher concentrations of NSC and soluble sugars to maintain the stability of biomembrane by osmotic adjustment under cold conditions and the survival over winter.

The distribution pattern of tree NSC may be influenced by several biological and environmental factors, including genetic variability, growth stage, density competition, light, water, nutrients, and temperature (Litton et al., 2004; Rachmilevitch et al., 2006; Dietze et al, 2014; Furze et al., 2019). This study focused on the effects of growth stage and tree age on NSC allocation. The concentrations and accumulations of soluble sugars and NSC and in the roots and stems remarkably changed between growth stages. Notably, the NSC allocations of roots and stems remarkably decreased from the dormancy stage to the fruit ripening stage, and the value in the roots is higher than that in the stems in both stages (**Figure 2**; **Table 3**, **4**). These results are consistent with the findings of Liu et al. (2018). As indicated by the relatively high levels of NSC, the roots of oil peony have substantial storage function compared with olives (Bustan et al., 2011). The seasonal variations of NSC further suggest that the roots and stems serviced as source structures at the early growth stage of Fengdan, similar to that of other deciduous tree species (Peng et al., 2012). This finding supports that NSC is the primary resource supply (Kozlowski, 1992; Pan et al., 2002). The contents of NSC and its components in the roots were significantly higher than those in other organs of Fengdan (**Tables 3**, **4**), indicating that the roots were the main storage structures (Gaucher et al., 2005). The contents of soluble sugars, starch, and NSC in each organ of Fengadan were in the range of 5.76%–17.47%, 2.86%–28.18%, and 13.95%–45.65%, respectively (**Tables 3**, **4**). These values were significantly higher than those of another shrub tree oil crop oil tea (0.26%–4.04%, 0.31%–2.75%, and 0.55%–6.43%) (Hao et al., 2020). This difference is mainly attributed to species specificity. Fengdan is a perennial subshrub, and its degree of lignification might be much lower than that of oil tea, holding relatively higher soluble sugars concentration. Nevertheless, both shrubs contained the highest NSC content in the seeds but lower soluble sugars content compared with the starch content. This phenomenon is related to the continuous consumption of soluble sugars in the process of seed development and oil storage.

No consistent trends of the seasonal variations of soluble sugars, starch, and NSC contents and accumulations were observed with the increase in Fengdan ages, indicating the absence of age effects. However, soluble sugars and NSC in the roots of 4a–6a plants were higher during dormancy than fruit ripening but lower in the 7a–9a roots (**Table 6**), indicating the existence of age effects. These differences also reflect the slight modulation of growth strategies from the younger to older Fengdan through the NSC allocation within plants. The roots of younger plants act as both sink and source structures, whereas those of the older ones act as sink structures. Considering that these metabolites in the stems decreased from dormancy to fruit ripening across all the age plants, the stems perform source functions aside from acting as sink structures and play a pivotal role as resource supply for the growth recovery of adult Fengdan plants (age ≥ 7a). These findings demonstrate that the age effects are expressed through the dynamic changes of NSC and the adjustments of sink-source functions of different organs.

### Correlation of NSC with biomass and yield

Understanding the correlation of NSC with yield is important for enhancing yield success (Zwieniecki et al, 2022). Allometric growth simulation analysis showed an allometric relationship between seed biomass and total accumulations of soluble sugars, starch, and NSC in most age Fengdan plants with an allometric growth index (slope) *b* < 1. Therefore, the NSC suppling vegetative growth outweighs yield formation. More importantly, the individual yield (seed mass) of Fengdan is generally correlated with the concentrations and accumulations of soluble sugars, starch, and NSC of each organ (**Table 7**). This finding supports that the level of NSC reserve play an important role in the yield formation of tree crops (Zwieniecki et al, 2022) but is inconsistent with the finding in oil olive that NSC reserves was not a yield determinant (Bustan et al., 2011). The positive correlation between yield and NSC (including soluble sugars and starch) accumulations of Fengdan plants during dormancy reflects that the roots and stems both are storage structures and suggests that NSC reserves should be replenished during the short post-harvest period prior to senescence to assure adequate reserves for bloom (Khezri et al., 2020; Zwieniecki et al., 2022). A positive correlation between yield and NSC was observed during fruit ripening. This pattern indicates that during the most active period, the reproductive NSC sink does not take precedence over reserve formation. It also hints that yield formation mainly depends on the photosynthesis in the current season.

Despite the general relationships mentioned above, evident differences were observed in the magnitude and temporal patterns of the correlations of NSC levels with the yield between the plants with different ages. The inverse relationship between yield and summer NSC indicates the expense of NSC reserve caused by yield formation (Zwieniecki et al, 2022). The stems are competitive sink structures with yield, because the levels of soluble sugars in the stems of all age plants and that of NSC in the stems of 4–6a plants during fruit ripening (i.e., in summer) were negatively correlated with yield. The negative relationships between the levels of NSC during dormancy and yield suggest that high NSC levels just prior to and during bloom are the most important prerequisite for achieving higher yields. Considering that NSC consists of soluble sugars and starch, the negative correlation between NSC and yield of Fengdan plants is mainly attributed to soluble sugars and yield. In addition, a high NSC content was achieved mainly by the influx of sugars from more distal sources during bloom. Hence, the concentration of soluble sugars in stems is the key element to yield success and can help project yield and provide information for the assessment of irrigation and fertilization needs. A slight difference between the younger (4–6a) and adult (7–9a) Fengdan plants is that the levels of NSC in the roots of later did not correlate with yield. This finding further suggests that, aside from age effects, stem is the key structure that adjust the sink-source relationship.

## Conclusion

Results show that tree age significantly impacts biomass and its allocation, NSC levels, especially the yield of Fengdan plants. The variations in the relationships between yield and seasonal fluctuations of NSC and biomass indicate that roots act as the key storage structures, whereas stems serve as sink and/or source functions for adult (7–9a) plants, the concentration of soluble sugars in stems is the key factor influencing Fengdan yield. These findings provide new insights into the mechanisms underlying the yield formation of Fengdan and have implications for manipulating sink-source relationship to achieve high yield.

## Data availability statement

The original contributions presented in the study are included in the article/supplementary material. Further inquiries can be directed to the corresponding author.

## Author contributions

CW, XM, and QL performed the wet lab work. CW performed the statistical analyses and wrote the first draft of manuscript. ZS, JY and YH conceived the study, participated in the design of the study, and finalized the manuscript. All authors contributed to the article and approved the submitted version.
